# Chemical and Biochemical Mechanisms Underlying the Cardioprotective Roles of Dietary Organopolysulfides

**DOI:** 10.3389/fnut.2015.00001

**Published:** 2015-02-02

**Authors:** Restituto Tocmo, Dong Liang, Yi Lin, Dejian Huang

**Affiliations:** ^1^Food Science and Technology Programme, Department of Chemistry, National University of Singapore, Singapore, Singapore; ^2^National University of Singapore (Suzhou) Research Institute, Jiangsu, China

**Keywords:** *Alliums*, stinky beans, dietary organopolysulfides, hydrogen sulfide, cardioprotection

## Abstract

Foods that are rich in organosulfides are highly regarded for their broad range of functions in disease prevention and health promotion since ancient time yet modern scientific study, particularly clinical studies could not agree with traditional wisdom. One of the complexities is due to the labile nature of organosulfides, which are often transformed to different structures depending on the processing conditions. The recent evidence on polysulfides as H_2_S donors may open up a new avenue for establishing structure and health promotion activity relationship. To put this development into perspective, we carried out a review on the recent progress on the chemistry and biochemistry of organopolysulfides with emphasis on their cardioprotective property. First, we briefly surveyed the foods that are rich in polysulfides and their structural diversity. This is followed by in-depth discussion on the chemical transformations of polysulfides under various processing conditions. We further reviewed the potential action mechanisms of polysulfides in cardioprotection through: (a) hydrogen sulfide releasing activity; (b) radical scavenging activity; and (c) activity in enzyme inhibition and intervention of gene regulation pathways. Based on the literature trend, we can conclude that the emerging concept of organopolysulfides as naturally occurring H_2_S donors is intriguing and warrants further research to establish the structure and activity relationship of the organopolysulfides as H_2_S donors.

## Introduction

According to the World Health Organization data, cardiovascular disease (CVD) is the number one cause of deaths and there will be estimated 23.3 million people who will die due to CVD by 2030 globally, which is more than the total population of Australia (1). Finding effective means for reducing the risk factors of CVDs has been a long-standing research theme for decades and much progress has been made and healthy diet is critically important. Fruits and vegetable are the key components of a healthy diet in part because they are rich in bioactive phytochemicals such as polyphenolic antioxidants, anti-inflammatory agents, and poly unsaturated fatty acids, particularly omega-3 fatty acids. For example, flavanols are shown to improve endothelium-mediated vasodilation (2) and European Food Safety Authority has approved the claim that consumption of 200 mg flavanols from cocoa daily can improve blood flow (3). In addition, cumulative evidence has suggested that dietary organosulfur compounds have a wide range of bioactivity, particularly cardiovascular health (4).

**Figure 1:**
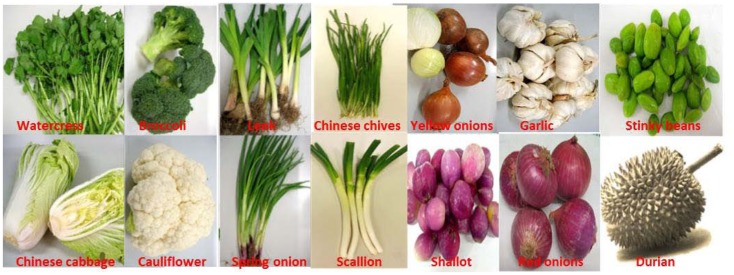
**Organosulfide-rich fruits and vegetables**.

Cruciferous vegetables and the *Allium* family are known for their rich contents of bioactive organosulfur compounds (Figure 1). Cruciferous vegetables are rich in glucosinolates, which undergo hydrolysis by thioglucosidase (myrosinase) to isothiocyanates (5) including sulforaphane in the broccoli (6), benzyl isothiocyanate in garden cress (7), and phenyl-ethyl isothiocyanate in watercress (8). While isothiocyanates from cruciferous vegetables have received great attention because of their potential anti-cancer activity through modulation of phase II enzyme activities (9, 10), the organosulfides in *Alliums* are well known for their broad spectrum of health promoting benefits, including anti-microbial, anti-cancer, and cardioprotective effects (4, 11). Yet, there is lack of consistent human clinical evidence to support the traditional wisdom. The chemistry of dietary organosulfur compounds is particularly complex because of their sensitivity to structural transformation mediated by the enzymes in the vegetables or during food processing. Consequently, it is a challenge to establish structure and bioactivity relationships. The bioactivity of allicin from garlic has been extensively studied and reviewed. However, human clinical trials in garlic found that allicin has no effect on reducing cholesterol level (12). The other important organosulfides in *Alliums* are volatile polysulfides readily formed when garlic is processed. They have shown potential for cardiovascular health promotion. Of all the *Allium* species currently known, garlic (*Allium sativum*) and onion (*Allium cepa*) are the most popular species due to various health benefits associated with their consumption. Garlic and onions have been greatly valued for their medicinal uses throughout the history of civilization. A large amount of literature have shown that the organosulfur compounds in these two species are associated with their biological functions against chronic ailments, including cancer, diabetes, and CVDs (4, 11). Yet, the labile chemistry of dietary organosulfides makes it challenging to establish structure and activity relationship. In this review, we summarized recent literatures regarding the organopolysulfide chemistry, biochemistry, and potential action mechanisms for promoting cardiovascular health, particularly as natural donor of H_2_S. Based on this, the future research directions of dietary organopolysulfides are suggested.

## Brief Survey of Organosulfides Rich Foods

**Figure 2:**
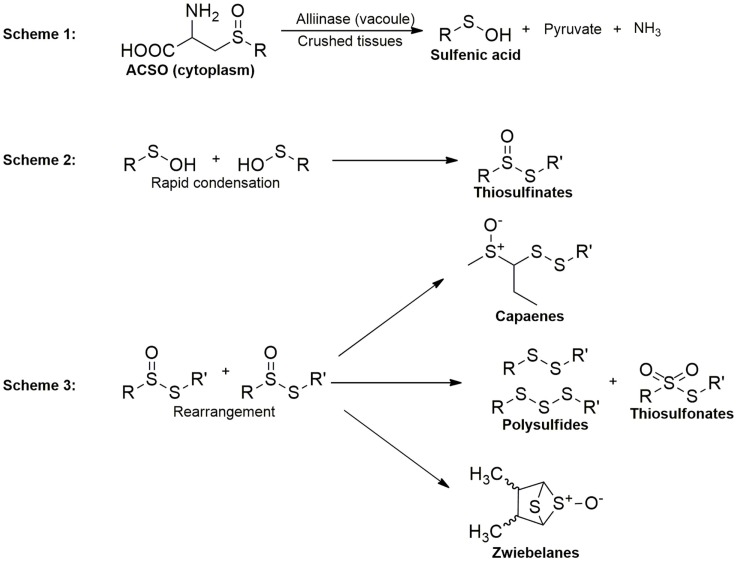
**Overview of organosulfide formation in *Alliums***.

The organosulfides in *Alliums* are classified into two major groups: (1) oil-soluble polysulfides and (2) water-soluble thiosulfinates, the intermediate formed upon the reaction of the vacuolar enzyme alliinase with the non-volatile *S*-alk(en)yl-l-cysteine-sulfoxides (ACSOs) present in the cell cytoplasm when *Alliums* are crushed. Thiosulfinates from garlic and onions are known for their wide biological activities, including antithrombotic, antihypertensive, antioxidant, antibacterial, and antifungal effects and these biological properties have been reviewed elsewhere (13, 14). Organopolysulfides (di-, tri-, and tetrasulfides) are the major OSCs in the oil-soluble components of *Alliums*. The formation of these lipophilic compounds starts with the alliinase-ACSO reaction, which produces highly unstable intermediates sulfenic acids, pyruvate, and ammonia. Condensation of sulfenic acids leads to the formation of thiosulfinates, which undergo further rearrangements into polysulfides and other OSCs, including cepaenes and zwiebelanes (Figure 2) (15). Alliinase is the key enzyme that facilitates the formation of the oil-soluble OSCs. Due to the compartmentalization of this enzyme and the ACSOs, cell rupture by cutting or maceration is necessary to facilitate their release (16) for the reaction to take place.

Aside from glucosinolates and isothiocyanates, *S*-methyl-l-cysteine sulfoxide (MCSO, or metthiin), is present in vegetables of genus *Brassica* (17). MCSO significantly contributes to the typical spicy and pungent aromas of culinary processed *Brassica* (18). Similar to *Alliums*, enzymatic catabolism of MCSO in *Brassicas* generates other sensory-active sulfur compounds including dimethyl thiosulfonate, dimethyl thiosulfinate, and dimethyl sulfides (19). The enzyme responsible, termed cystine lyase (EC 4.4.1.8), behaves similarly as the alliinase in garlic, except that it can also hydrolyze l-cystine (20). Hydrolysis of MCSO generates highly reactive methyl sulfenic acid, which condenses to generate methyl methanethiosulfinate. Subsequently, thermal degradation of methyl methanethiosulfinate forms volatile polysulfides, majority of which are composed of dimethyl disulfide and dimethyl trisulfide (18). The occurrence, concentration, and distribution of MCSO in cruciferous vegetables are well documented (18, 19, 21, 22) and its biological functions have recently been reviewed in Ref. (23). In general, MSCO is found at about 1–2% dry weight in vegetables that belong to *Brassicaceae*, especially those of the genus *Brassica* (24). Although MCSO is universally present in *Brassicas*, factors associated to species and varietal differences influence the concentration and distribution of MCSO in these plants (23). Other factors affecting MCSO concentrations include environmental conditions, nutrient availability, harvest timing, and storage practices (25–27). While is it generally accepted that MCSO and other thermally generated breakdown products such as *S*-methyl methanethiosulphinate and *S*-methyl methanethiosulfonate, contribute to the typical flavor of processed cruciferous vegetables, their cardiovascular effect, although limited, has been reported (28, 29).

Garlic has been used for centuries as a traditional remedy to treat infectious diseases (30, 31). One of the main sulfur-containing compounds present in raw garlic is γ-glutamylcysteine. It has been proposed that γ-glutamylcysteine, along with glutathione are the starting compounds, which undergo hydrolysis and oxidation leading to the biosynthesis of ACSOs (13). In garlic, *S*-allyl-l-cysteine sulfoxide (alliin) is the predominant ACSO, while *S*-methyl-l-cysteine sulfoxide (methiin) and *S*-propenyl-l-cysteine sulfoxide (isoalliin) are present in smaller amounts (32). Another major compound formed upon hydrolysis of garlic is *S*-allylcysteine (SAC), a water-soluble thiosulfinate produced during aqueous garlic extraction catalyzed by the enzyme γ-glutamyl transpeptidase (33). Some of the health-beneficial functions of garlic are attributed to its organosulfur components, including the main ACSO precursor alliin and allicin, a thiosulfinate resulting from the lyses of alliin by alliinase. However, allicin is a transient compound that rapidly undergoes non-enzymatic decomposition into numerous oil-soluble polysulfides such as diallyl monosulfide (DAS), diallyl disulfide (DADS), diallyl trisulfide (DATS), diallyl tetrasulfide, and allyl methyl trisulfide (34, 35). The main components of garlic oil (Table 1) obtained by steam and hydrodistillation are DADS, DATS, allyl methyl trisulfide, and 2-vinyl-4H-1,3-dithiin (36–40). All eight thiosulfinates containing combinations of methyl, 1-propenyl, and 2-propenyl substituents are present in garlic homogenates, which explains the presence of lipid-soluble polysulfides with similar substituent combinations in distilled garlic oil (41).

**Table 1:** **Acyclic and cyclic organopolysulfides from the oil-soluble components of common dietary sources**.

Organosulfur compound	Structure	Source	Reference
**ACYCLIC DISULFIDE**			
Diallyl disulfide	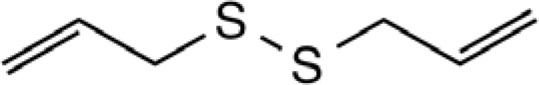	Garlic, shallot, onion, leek, Chinese chive, rakkyo	(42–48)
Dipropyl disulfide	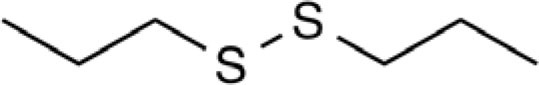	Onion, shallot, leek, scallion, Welsh onion, rakkyo	(42, 45, 48–52)
Dimethyl disulfide	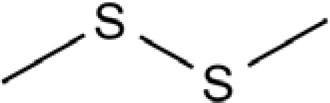	Shallot, garlic, scallion, Welsh onion, Chinese chive, rakkyo	(43, 44, 46, 47, 49–51, 53)
Diethyl disulfide	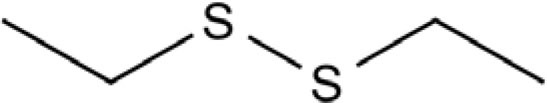	Durian	(54–58)
Allyl methyl disulfide	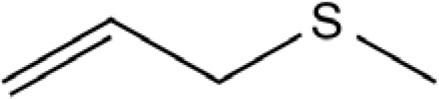	Garlic, Chinese chive, Rakkyo	(40, 44, 46, 47, 53)
Allyl propyl disulfide	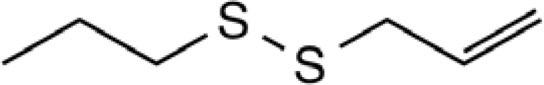	Garlic	(53)
Methyl propyl disulfide	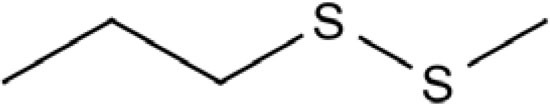	Leek, onion, shallot, scallion, Welsh onion, Rakkyo, Chinese chive	(42–45, 49–51)
Methyl ethyl disulfide	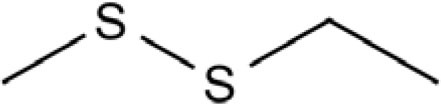	Durian, Rakkyo, Chinese chive	(44, 59)
Ethyl propyl disulfide	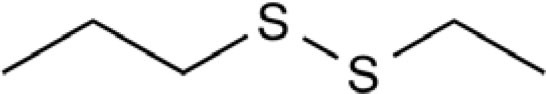	Durian, onion	(49, 59)
Methyl 1-propenyl disulfide	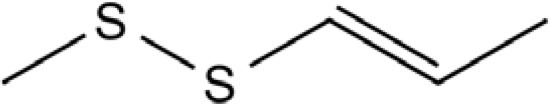	Leek, garlic, onion, shallot, scallion, Welsh onion, Chinese chive, rakkyo	(40, 42–46, 49–51, 53)
Ethyl 1-propenyl disulfide	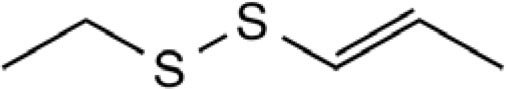	Rakkyo, Chinese chive	(44)
Allyl 1-(E)-propenyl disulfide	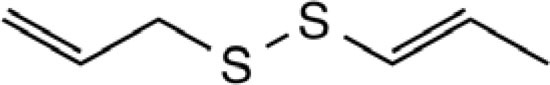	Chinese chive	(46)
Propyl 1-propenyl disulfide	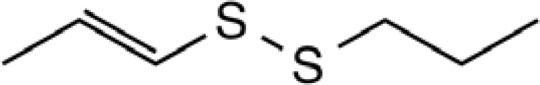	Leek, onion, shallot, scallion, Welsh onion, Chinese chive, rakkyo	(42–45, 49–51)
**ACYCLIC TRISULFIDE**			
Diallyl trisulfide	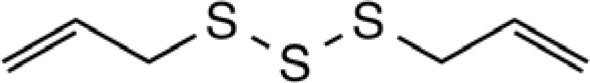	Garlic, rakkyo, Chinese chive	(40, 44, 47, 53)
Dimethyl trisulfide	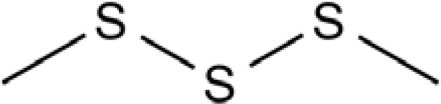	Leek, garlic, shallot, onion, scallion, Welsh onion, Chinese chive, rakkyo	(42, 43, 45, 46, 49–52)
Dipropyl trisulfide	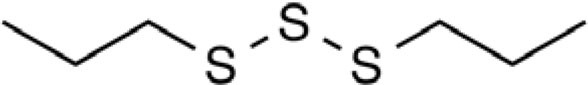	Leek, shallot, Chinese chive, onion, rakkyo	(42, 44, 45, 48, 49, 52)
Diethyl trisulfide	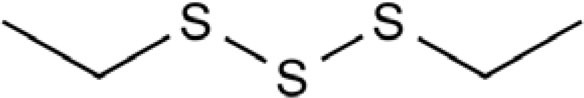	Durian	(54–57, 59)
Allyl methyl trisulfide	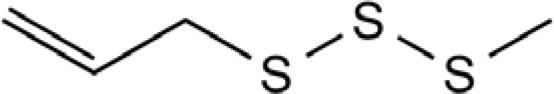	Garlic, Chinese chive, rakkyo	(40, 44, 46, 47, 51, 53)
Methyl propyl trisulfide	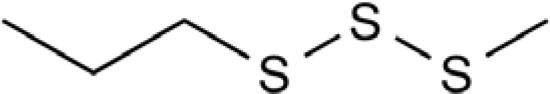	Onion, shallot, Chinese chive, rakkyo	(42–44, 49, 51, 52)
Ethyl methyl trisulfide	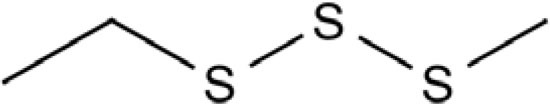	Rakkyo, Chinese chive	(44)
Methyl 1-propenyl trisulfide	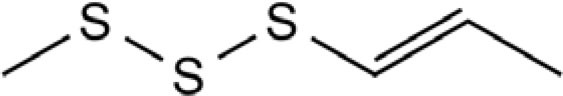	Onion, shallot, Rakkyo, Chinese chive	(42–44, 51)
Allyl 1-propenyl trisulfide	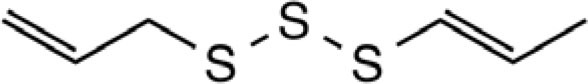	Garlic, rakkyo	(51)
Propyl 1-propenyl trisulfide	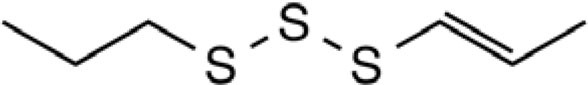	Onion, shallot, Chinese chive	(42–44, 49)
**ACYCLIC TETRASULFIDE**			
Dimethyl tetrasulfide	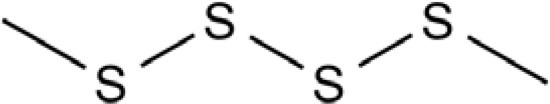	Shallot, onion, rakkyo, Chinese chive	(43, 44, 51)
Diallyl tetrasulfide	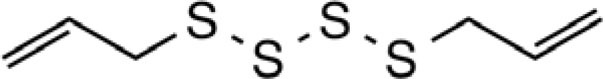	Garlic	(40)
Propyl 1-propenyl tetrasulfide	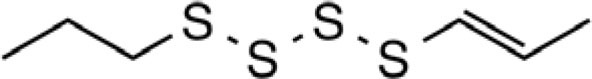	Chinese chive, rakkyo	(44)
Methyl pentyl tetrasulfide	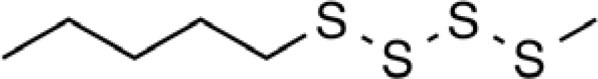	Chinese chive	(44)
Dipropyl tetrasulfide	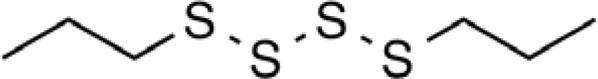	Chinese chive, leek	(44, 48)
Allyl propyl tetrasulfide	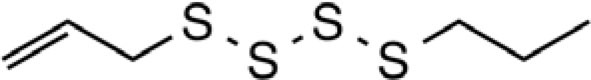	Chinese chive	(44)
**CYCLIC POLYSULFIDES**			
3-Vinyl-[4H]-1,2-dithiin	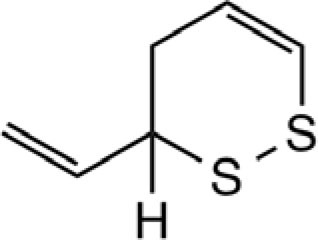	Garlic, Chinese chive	(46, 51, 53)
2-Vinyl-[4H]-1,3-dithiin	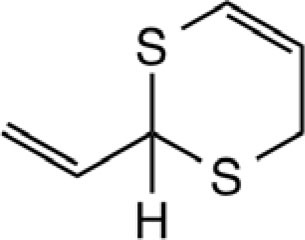	Garlic, Chinese chive	(46, 51, 53)
[3H,4H]-1,2-dithiin	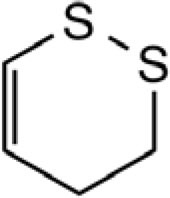	Chinese chive	(46)
[2H,4H]-1,3-dithiin	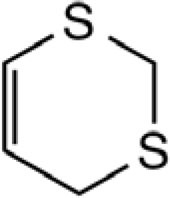	Chinese chive	(46)
3,5-Diethyl-1,2,4-trithiolane	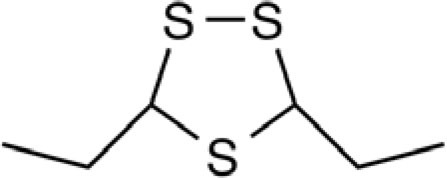	Leek, garlic, scallion, Welsh onion, rakkyo	(45, 50, 51, 53)
3,5-dimethyl-1,2,4-trithiolane	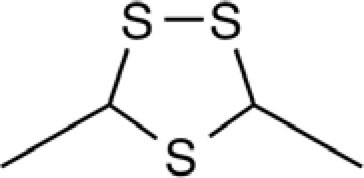	Durian, shallot	(52, 59)
3-Methyl-5-ethyl-1,2,4-trithiolane	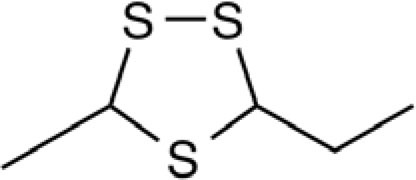	Scallion, Welsh onion, rakkyo	(50)
3-Ethyl-1,2-dithi-4-ene	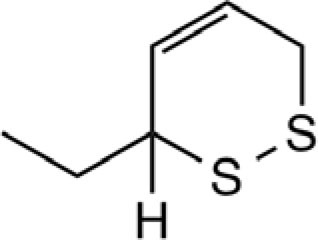	Onion, shallot	(43, 49, 52)
3-Ethyl-1,2-dithi-5-ene	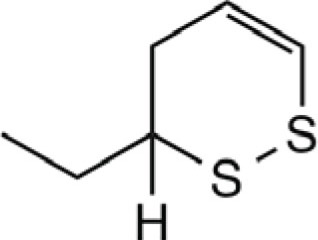	Onion, shallot	(43, 52)

The organosulfide profile of onion oil includes polysulfides with different combinations of methyl, propyl, and 1-propenyl substituents, except for the compound with 1-propenyl and 1-propene substituents on either side of the -S-S(=O)- group of a thiosulfinate (60). 1-Propenyl 1-propenethiosulfinate (CH_3_-CH =CH-SS(O)CH =CH-CH_3_) has not been detected in *Allium* tissues possibly due to its oxidation to bissulfine or cyclization to zwiebelanes and a possible reaction with sulfenic acids to form cepaenes (15, 32).

Distilled oils and solvent extracts from other *Allium* species are also known for their predominantly high contents of organosulfur compounds. These species include Chinese chive (*Allium tuberosum* Rottl. ex.), leek (*Allium porrum* L.), shallot (*Allium cepa* L. Aggregatum Group), rakkyo (*Allium chinense* G. Don), scallion (*Allium fistulosum* L. var caispitosum), and Welsh onions (*Allium fistulosum* L. var maichuon) (Figure 1). Summarized in Table 2 are the concentrations of the various oil-soluble polysulfides from their respective sources. Apparently, the abundance of the individual compounds depends on which *Allium* species they are found, although it is well known that garlic oil is rich in allyl polysulfides while onion oil is typically characterized by their high amounts of polysulfides with propyl substituents. While varietal differences may have effects on the levels of polysulfides, it is rather premature to conclude on this yet due to limited literature available.

**Table 2:** **Relative abundance of organopolysulfides from common dietary sources**.

Organosulfur compound	Source	Concentration (GC% area)
**ACYCLIC DISULFIDE**		
Diallyl disulfide	Garlic	13.07^1^, 29.10^2^, 28.40^3^
	Chinese chive	0.70^4^
Dipropyl disulfide	Onion	1.18^5^
	Shallot	4.16^6^
	Scallion	8.81^7^, 9.79^8^
	Welsh onion	0.47^7^, 4.28^8^
	Chinese chive	5.50^4^
Dimethyl disulfide	Garlic	2.20^3^
	Shallot	1.99^6^
	Scallion	0.04^7^, 0.24^8^
	Welsh onion	0.04^7^, 0.78^8^
	Rakkyo	15.00^4^
	Chinese chive	7.30^4^
Diethyl disulfide	Durian	5.15^9^, 2.17^10^
Allyl methyl disulfide	Garlic	1.7^20^, 9.10^3^
	Rakkyo	39.30^4^
	Chinese chive	4.80^4^
Methyl propyl disulfide	Garlic	0.20^3^
	Onion	1.07^5^
	Shallot	3.59^6^
	Scallion	0.78^7^, 1.41^8^
	Welsh onion	0.59^7^, 2.76^8^
	Rakkyo	1.30^4^
	Chinese chive	5.50^4^
Methyl ethyl disulfide	Durian	0.25^9^, 0.25^10^
Ethyl propyl disulfide	Durian	2.11^9^
Methyl 1-propenyl disulfide (cis/trans)	Garlic	0.40^2^
	Onion	0.72^5^
	Shallot	8.00^6^
	Scallion	0.02^7^, 1.51^8^
	Welsh onion	5.91^7^
	Rakkyo	2.90^8^
	Chinese chive	2.40^4^
Ethyl 1-propenyl disulfide	Rakkyo	1.70^4^
	Chinese chive	3.70^4^
Allyl 1-propenyl disulfide (cis/trans)	Rakkyo	9.50^4^
	Chinese chive	2.40^4^
Propyl 1-propenyl disulfide (cis/trans)	Shallot	7.22^6^
	Scallion	3.96^7^, 4.72^8^
	Welsh onion	0.20^7^, 0.78^8^
	Chinese chive	6.40^4^
**ACYCLIC TRISULFIDE**		
Diallyl trisulfide	Garlic	11.49^1^, 37.30^2^, 20.40^3^
	Rakkyo	0.90^4^
	Chinese chive	0.40^4^
Dimethyl trisulfide	Garlic	0.20^2^, 2.70^3^
	Onion	0.43^5^
	Shallot	18.81^6^
	Scallion	0.17^7^, 1.75^8^
	Welsh onion	0.28^7^, 6.14^8^
	Rakkyo	12.60^4^
	Chinese chive	6.0^4^
Dipropyl trisulfide	Shallot	5.55^6^
	Scallion	0.79^7^
	Welsh onion	1.81^8^
	Chinese chive	6.00^4^
	Durian	4.77^10^
Diethyl trisulfide	Durian	2.80^9^, 21.17^10^
Allyl methyl trisulfide	Garlic	10.40^2^, 17.50^3^
	Rakkyo	4.50^4^
	Chinese chive	3.40^4^
Methyl propyl trisulfide	Shallot	19.93^6^
	Scallion	0.28^7^, 12.75^8^
	Welsh onion	0.91^7^, 12.97^8^
	Chinese chive	9.90^4^
Ethyl methyl trisulfide	Rakkyo	1.10^4^
	Chinese chive	0.20^4^
Methyl 1-propenyl trisulfide (cis/trans)	Onion	0.82^5^
	Shallot	4.85^6^
	Scallion	0.88^7^, 8.39^8^
	Welsh onion	1.43^7^, 3.56^8^
	rakkyo	1.30^4^
	Chinese chive	2.90^4^
Propyl 1-propenyl trisulfide	Shallot	9.97^6^
	Scallion	0.10^7^, 6.59^8^
	Welsh onion	4.03^7^, 2.94^8^
	Chinese chive	7.70^4^
**ACYCLIC TETRASULFIDE**		
Dimethyl tetrasulfide	Scallion	0.54^7^, 0.85^8^
	Welsh onion	0.36^7^, 2.15^8^
	Rakkyo	1.10^4^
	Chinese chive	3.20^4^
Diallyl tetrasulfide	Garlic	3.00^2^, 0.70^3^
Propyl 1-propenyl tetrasulfide	Scallion	0.49^8^
	Welsh onion	0.24^8^
Dipropyl tetrasulfide	Scallion	0.15^7^, 1.11^8^
	Welsh onion	2.00^7^, 0.82^8^
	Chinese chive	1.00^4^
Allyl propyl tetrasulfide	Chinese chive	1.30^4^
**CYCLIC POLYSULFIDES**		
3-Vinyl-[4H]-1,2-dithiin	Garlic	32.70^3^
2-Vinyl-[4H]-1,3-dithiin	Garlic	43.90^3^
[3H,4H]-1,2-dithiin	Garlic	1.00^2^
[2H,4H]-1,3-dithiin	Garlic	1.85^2^
3,5-Diethyl-1,2,4-trithiolane	Garlic	1.00^1^, 0.50^3^
	Welsh onion	11.39^8^
	Rakkyo	8.42^8^
3,5-Dimethyl-1,2,4-trithiolane	Durian	2.30^9^, 3.91^10^
3-Ethyl-1,2-dithi-4-ene	Onion	0.76^5^
3-Ethyl-1,2-dithi-5-ene	Onion	0.70^5^
1,2,4-trithiolane	Stinky bean	50.68^11^, 4.75^12^
1,3,5-trithiane	Stinky bean	0.21^12^
1,2,4,5-tetrathiane	Stinky bean	2.53^11^, 0.34^12^
1,2,4,6-tetrathiepane	stinky bean	11.21^11^
1,2,3,5,6-pentathiepane	stinky bean	3.78^11^

*References: ^1^(38); ^2^(40); ^3^(37); ^4^(44); ^5^(61); ^6^(42); ^7^(50); ^8^(62); ^9^(59); ^10^(58); ^11^(63); ^12^(64)*.

*GC Column used: ^1^SE-52 on Chromosorb B; ^2,3^HP-5MS capillary column; ^4^DB-1 fused silica capillary column; ^5^DB-5 fused silica column; ^6^SCOT Carbowax 20M; ^7,8^HP-1 fused silica capillary column; ^9^DB-FFAP column; ^10^SPB-5 column; ^11^SPB-1 column; ^12^TC-WAX capillary column*.

Shallot is an important *Allium* species commonly used in many Asian diets. High amounts of organosulfides have been reported in the distilled and solvent extracted oils of shallot (42). Data from our lab also shown that hydrodistilled oil from shallot originating from Vietnam contain high amounts of onion-type polysulfides (52). Distilled oil and solvent extracts of Welsh onions and scallions consists 82–87% of organosulfides (50, 62). Similarly, organosulfides from the essential oils of Chinese chive and rakkyo comprised 88–94% of their total volatiles (44). Twelve varieties of solvent extracted oil from Chinese chive were found to contain disulfides, trisulfides, and vinyldithiins formed enzymatically from methiin as their precursor (46). The individual polysulfides in these less popular *Allium* species are listed in Table 1.

**Figure 3:**
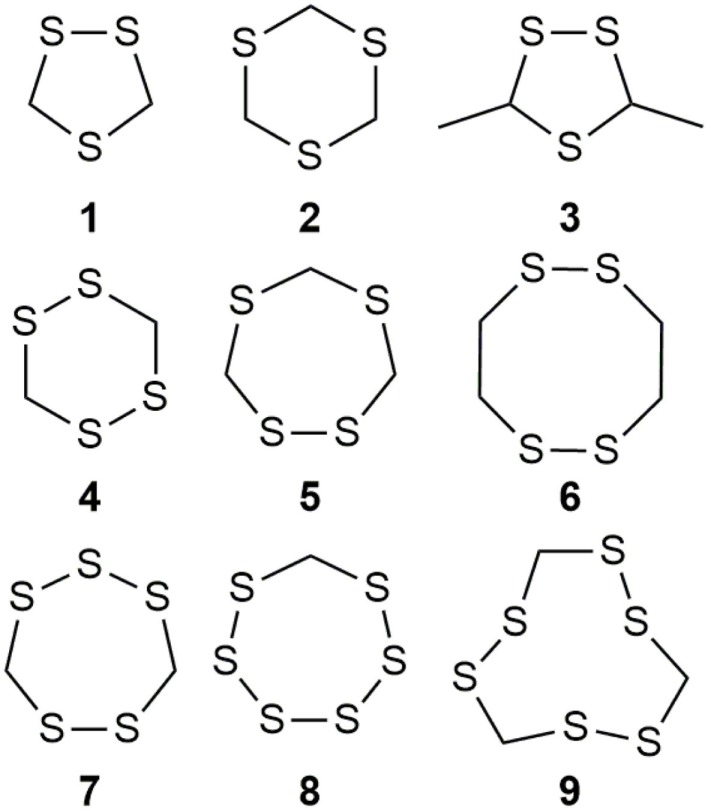
**Cyclic organopolysulfides from stinky bean**. **1**, 1,2,4-trithiolane; **2**, 1,3,5-trithiane; **3**, 3,5-dimethyl-1,2,4-trithiolane; **4**, 1,2,4,5-tetrathiane; **5**, 1,2,4,6-tetrathiepane; **6**, 1,2,4,5-tetrathiocane; **7**, 1,2,3,5,6-pentathiepane (lenthionine); **8**, 1,2,3,4,5,6-hexathiepane; **9**, 1,2,4,5,7,8-hexathionane.

Organosulfur compounds are also present in the seeds of *Parkia speciosa* Hassk, commonly known as “petai” or stinky bean because of its unpleasant smell. In addition to its culinary uses, stinky bean is believed to have anti-microbial (65, 66), antioxidant (67–69), hypoglycemic (70), antiulcer (71), and antihypertensive (69) effects. Cyclic polysulfides are the major components of cooked petai (63). Earlier reports indicate that cyclic polysulfides [1,2,4-trithiolane (**1**), 1,3,5-trithiane (**2**), 1,2,4,6-tetrathiepane (**5**), and 1,2,3,5,6-pentathiepane (lenthionine) (**7**), 1,2,4,5,7,8-hexathionine] were the major constituents of stinky bean (Figure 3) (65, 68). In another study, hydrogen sulfide was found as the most abundant (41.3%) headspace constituent of stinky bean (64). Other cyclic polysulfides that have been found from stinky bean, include 3,5-dimethyl-1,2,4-trithiolane (**3**), 1,2,4,5-tetrathiane (**4**), 1,2,4,5-tetrathiocane (**6**), 1,2,3,4,5,6-hexathiepane (**8**), and 1,2,4,5,7,8-hexathionane (**9**).

Of the edible fruits, durian (*Durio zibethinus* Murray) is perhaps the only tropical fruit known for its organosulfur contents. Durian, dubbed as the king of tropical fruits, is an exotic fruit with extensive popularity in Southeast Asia due to its distinct taste, odor, and texture. Of the 108 volatiles compounds determined in durian, 18 organosulfides were identified, making sulfurous compounds the second major volatile constituents, after ester group (56). Durian from Indonesia was reported to contain 17 (55) and 43 (72) organosulfides in two individual studies, with some common organosulfides detected such as *S*-ethyl thioacetate, diethyl disulfide, and 3,5-dimethyl-1,2,4-trithiolane. The volatile constituents of three different durian varieties D15, D28, and D74 were compared and it was found that D28 has the highest organosulfides content (54). In a separate study, organosulfides were the predominant compounds in durian in terms of quantity, contributing to more than 50% of the total volatiles (57).

Overall, there are various natural organosulfide sources including durian, the *Allium* vegetables, and stinky bean with characteristically high amounts of organosulfides. The formation of garlic- and onion-type OSCs follow a similar reaction mechanism with the ACSO-alliinase reaction as the main step after *Alliums* are crushed. Except for garlic and onions, the biological functions of the OSCs from these dietary sources are not yet fully explored.

## Transformations of Dietary Organosulfides Under Different Processing Conditions

**Figure 4:**
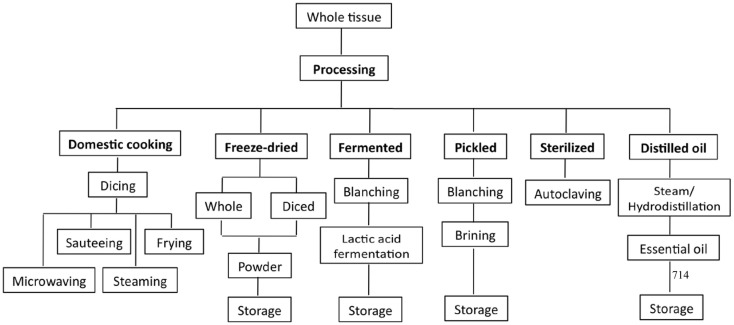
**Overview of the different processing methods for *Allium* products**.

Unlike fruits, vegetables are commonly cooked before they are consumed, except in the case where they are added as ingredients to salads. In general, cooking methods that involve heating (i.e., boiling, steaming, and microwaving) are commonly used to prepare homemade dishes. The most common commercially available form of *Allium* products is the raw form. However, other forms of *Allium* products have increased their popularity and availability in the international market (11). Common *Allium* products available in the market include powdered or dried garlic flakes, garlic and onion essential oils, naturally fermented garlic or aged garlic extract (AGE), pickled fermented and unfermented garlic, and pastes (73). An overview of the various processed forms of *Allium* products is shown in Figure 4.

**Figure 5:**
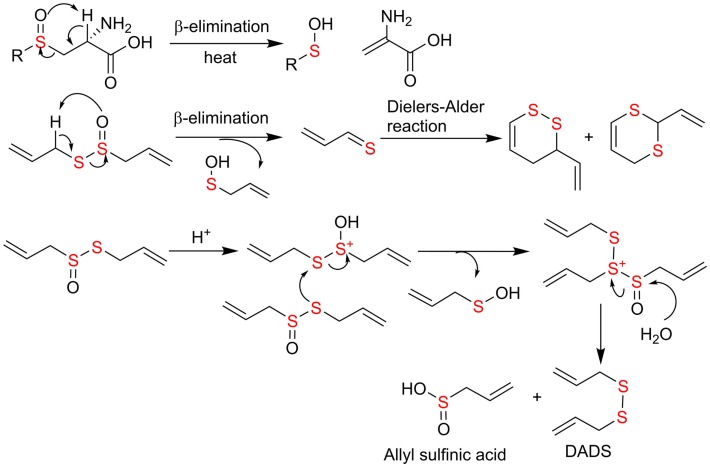
**Proposed mechanism of chemical transformation from ACSO to organosulfides through elimination and acid-mediated nucleophilic substitution reactions**.

The study on the chemical transformation of single organosulfide would shed some light on the key reaction principles and the factors governing the transformation in complex food matrix. Sulfoxides are known to undergo β-elimination reaction upon heating and this property has been applied in organic synthesis of olefins sulfenic acids as by-products (74). In principle, ASCOs can form sulfenic acid through thermal elimination perhaps at higher temperature cooking in food matrix. However, this has not been reported in the literature yet. Instead, the focus is on alliinase catalyzed β-elimination reaction at room temperature. The resulting sulfenic acid quickly dehydrates to form allicin, which may undergo β-elimination again to regenerate sulfenic acid and 2-propenethial. The latter leads to the formation of cyclic sufides through Diels–Alder reaction (75). Formation of disulfides and polysulfides from allicin was proposed to be mediated by acid (76) (Figure 5). Beta-elimination from methyl substituted sulfoxide, *S*-methyl-l-cysteine sulfoxide, were shown to give rise to primarily dimethyl disulfides, which are responsible for the characteristic flavors of *Alliums* (18). Cyclic sulfides from stinky beans are not as common. Chemically, they are likely to be formed from formaldehyde and hydrogen sulfide followed by oxidation. In biological system, their formation mechanisms remain to be elucidated (63–65).

**Figure 6:**
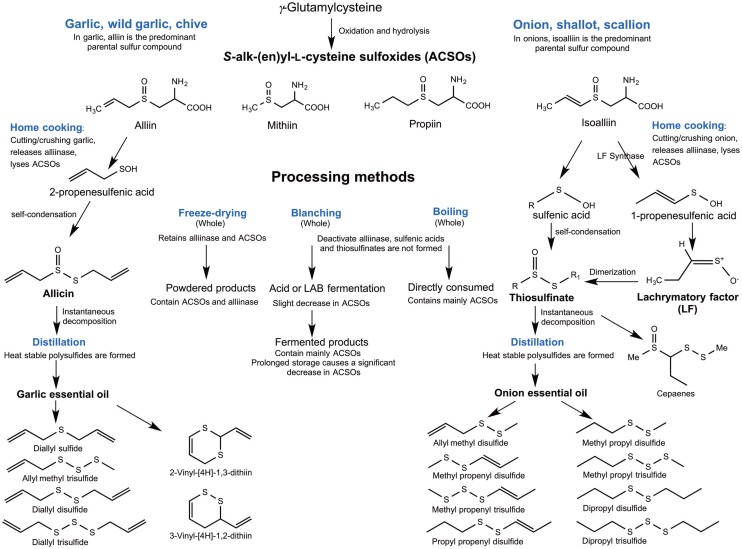
**Overview of the transformations of organosulfur compounds in *Alliums* during processing**.

In home cooking, cloves or whole onions are often cut into small pieces or crushed into pastes. Biotransformation of organosulfides in *Alliums* begins when cytoplasmic ACSOs are released and mixed with the vacuolar alliinase. When garlic cloves or onion are cooked as whole, deactivation of the alliinase would limit the transformation of ACSOs, whereas fresh macerates of garlic or onion would have high contents of thiosulfinates. Thiosulfinates were found to be present in *Allium* homogenate with stability lasting for 26 h under room temperature, except for considerable losses observed in those represented by MeCH = CHS(O)SR and MeS(O)SMe (60). Thiosulfinates can decompose to generate other form of organosulfides (i.e., linear polysulfides) (15). This decomposition is greatly affected by typical processing parameters such as temperature, pH, and storage time (77, 78). Commercial *Allium* products are available in the form of macerates (e.g., crushed garlic in oil) or homogenates, but the more commonly available ones are freeze-dried powder, essential oil, or cloves in fermented or acidified brine (11, 79). In garlic or onion essential oils, thermal decomposition of thiosulfinates generates various mono-, di- and trisulfides as a result of different distillation processes (11, 13). Indeed, GC-MS analysis of *Allium* essential oil has revealed predominance of mixed polysulfides (Table 1). Essential oils from *Alliums* are highly valued for their medical and biological functions because of their contents of organopolysulfides, including DADS, DATS, allyl methyl trisulfide, diallyl tetrasulfide in garlic distilled oil and dipropyl disulfide, dipropyl trisulfide, methyl propenyl disulfide, and methyl propyl trisulfide in distilled onion oil (11). An overview of the organosulfide transformations in *Alliums* as affected by various processing conditions is illustrated in Figure 6.

Aside from distilled oils, powdered form of garlic or onion is widely sold in the market. Freeze-drying is a major processing step employed in making this product. Since cloves or whole onions are typically used during freeze-drying, tissues remain intact; hence, hydrolysis of ACSOs by alliinase may not occur. Therefore, the powder basically has similar content of ACSO profile as the fresh ones. Freeze-drying of high-pressure processed (HPP) onion resulted in variable effects on the concentrations of major polysulfides (80). Significant decreases (*P* < 0.05) in dipropyl disulfide (59–82%), dipropyl trisulfide (62–85%), and trans-propenyl propyl disulfide (38–65%) were observed, retaining the levels of dimethyl trisulfide and methyl 1-propenyl disulfide. In our recently published work, we found that freeze-drying preserved most of the individual organosulfides in shallot, although total organosulfur compounds decreased due to reduction in the concentrations of only a few compounds (52). Moreover, freeze-drying showed retention of polysulfides comparable with other drying methods (air- and over-drying) (40). These observations indicate that freeze-drying is a good alternative method in producing dried *Allium* products with comparable polysulfide contents as those of the fresh.

Other drying methods have also been shown to have direct effects on the organosulfides of different *Allium* species. The effects of microwave drying and cabinet drying on the volatile components of hydrodistilled oil derived from garlic powder have been compared (81). DADS and diallyl tetrasulfide increased with both drying methods, however, DATS and allyl methyl trisulfide decreased, with microwave drying showing greater reduction. The observed reduction of polysulfides with microwave drying can be attributed to the effect of heat generated that might have driven off the volatiles and deactivated alliinase. It has been previously demonstrated that heating at 80°C for 10 min can partially deactivate alliinase (82).

The effect of long-term frozen storage (−20°C) of leek slices showed significant reduction of 14 sulfur compounds after 12 months (45). Some polysulfides including, propyl (E)-propenyl di- and trisulfides and dipropyl disulfide, increased after 2 weeks but compounds such as dimethyl disulfide and 2-propenyl disulfide were not detected after 4–6 months. The increase in concentration after 2 weeks of frozen storage implies that alliinase maintains its catalytic activity in sliced leeks even during 2 weeks of freezing.

Due to the sensitivity of alliinase to heat, boiled *Alliums* are expected to have no heat sensitive thiosulfinates, which have their purported bioactivity. For instance, the loss in the *in vitro* anti-aggregatory activity (IVAA) of garlic was suggested to be due to the sensitivity of thiosulfinates to heat after 10 min of boiling (100°C) (83). Similar results were observed in onion thiosulfinates. Boiling for 46 min completely suppressed IVAA, which the author correlates with the decrease in thiosulfinates (84). Minimal duration of boiling (<3 min) may preserve thiosulfinates as indicated by a maintained IVAA of both onion and garlic (83, 84). In another study, heat treatment by microwave (500 W) and convection oven (200°C) generally decreased thiosulfinate content as indicated by a reduction in IVAA (78). However, the extent to which these antiplatelet agents are destroyed varied depending on the method (whole, quarters, and crushed) of sample preparation with whole showing longer retention of IVAA (after 30 min) as compared to quarters (20 min) and crushed samples (10 min).

Among the *Allium* products in the market, there appears to be a fair acceptance of pickled blanched garlic, which comes as either fermented or unfermented. The main step in the process is blanching (90°C) of garlic cloves, which may deactivate alliinase and, therefore, have direct implication in the formation of other organosulfur compounds. The next step is brining whereby garlic cloves are immersed in an acidified brine containing lactic acid (85). Fermented pickled garlic involves subjecting the blanched garlic cloves to lactic acid bacteria fermentation, typically *Lactobacillus plantarum* or *Lactobacillus pentosus* (86). The final pH of the brine is around 3.9 with a titratable acidity of 1.2% (as lactic acid) (86). It was found that only SAC and GSAC slightly decreased after blanching (79). Therefore, blanching for 5 min is not that detrimental to the major OSC precursors in garlic such as alliin. However, after pickling and fermentation, the contents of alliin, isoalliin, and cycloalliin significantly decreased and became even much lower (23–28% loss) after 1 year of storage at refrigerated temperature (79). The blanching step is carried out mainly to deactivate alliinase. Consequently, the contents of oil-soluble polysulfides will be impacted by blanching. Pickling and fermenting garlic may eliminate the pungent flavor brought about by formation of thiosulfinates. Flavor improvement using these processing techniques could be useful as a marketing strategy. However, these processing methods may have critical implications on the formation of other bioactive organosulfur compounds in *Alliums*.

The sensitivity of organosulfides to processing methods used pose a challenge to processing *Alliums* so that the end products have optimal bioactive organosulfides. This would require research work that can delineate the structure and activity relationship through mechanistic studies.

## Action Mechanisms of Polysulfides: As H_2_S Donors

Perhaps the most interesting and promising aspects of dietary organosulfur compounds are their potentials to be precursors of hydrogen sulfide (H_2_S), a colorless, flammable, and toxic gas with the characteristic foul odor of rotten eggs. For quite a long time, H_2_S attracted attention mainly because of its toxicity, however, in the past decades H_2_S has been found as a gaseous signaling molecule, which plays important roles in many physiological and pathological conditions (87). H_2_S is the third and newest member of gasotransmitter family, along with nitric oxide (NO) and carbon monoxide (CO) (88, 89).

Since the discovery that endogenous H_2_S selectively enhance *N*-methyl-d-aspartate (NMDA) receptor (a glutamate receptor controlling synaptic plasticity and memory function) mediated response in 1996 (90), tremendous progress has been made in the understanding of H_2_S physiology. H_2_S is associated with a wide yet still expanding range of physiological events – their cardiovascular benefits attracting the most attention (87, 91). The first paper concerning the cardiovascular effect of H_2_S was published in 1997 (92), in which H_2_S was found to induce smooth muscle relaxation *in vitro*. Four years later it was reported that the H_2_S was a K_ATP_ channel activator, which was responsible for the vasorelaxation effect *in vivo* (93). Since then, the beneficial effects of H_2_S have been shown in many other studies (94). Hydrogen sulfide dilates blood vessel (95), protects against ischemia-reperfusion injury in myocardium (96), protects against heart failure by reducing oxidative stress, increasing myocardial vascular density, and preserving mitochondrial function (97). Furthermore, H_2_S prevents atherosclerosis by reducing smooth muscle cell proliferation and inhibiting adhesion molecule expression (98).

Hydrogen sulfide is produced endogenously in mammalian tissues mainly by enzymatic metabolism of l-cysteine. Currently, four enzymes, including cystathionine γ-lyase (CSE), cystathionine β-synthase (CBS), 3-mercaptopyruvate sulfur transferase (3-MST), and cysteine aminotransferase (CAT) have been found to be involved in its biological production (87, 99, 100). CSE and CBS are believed to be the major enzymes responsible for endogenous H_2_S synthesis. CSE has been reported in various organs including kidney, liver, uterus, and placenta but it is predominantly found in the vasculature and liver; while CBS is expressed in the liver and central nervous system.

Some non-enzymatic pathways that lead to H_2_S generation also exist, for example, the reaction between naturally occurring polysulfides and biological thiols, mainly through thiol-disulfide exchange reactions. The generation of H_2_S from the reaction between glutathione and calicheamicin γ1 (a natural antitumor agent with an allyl trisulfide group) was reported in 1994, when Myers et al. were trying to elucidate how calicheamicin γ1 initiated the DNA cleavage process (101). H_2_S was also generated upon the reaction between glutathione and 7-methylbenzopentathiepin, an analog of naturally occurring antibiotics varacin (102), however the H_2_S generation in these studies attracted little attention.

In 2007, Benavides et al. reported that DATS and DADS can be converted to H_2_S by human red blood cells or rat aorta ring, and the H_2_S produced exerts a vasorelaxant effect on rat aorta (103). Hence, they suggested that H_2_S mediates the vasoactivity of garlic. They also demonstrated that H_2_S production was through the reaction between DADS/DATS and glutathione. Since the conventional thiol-disulfide exchange between DADS and GSH does not produce H_2_S, they proposed that DADS undergo nucleophilic substitution at α carbon, producing the key intermediate allyl perthiol, which subsequently reacts with GSH to release H_2_S (103). They also found that the H_2_S releasing activity of organosulfurs from garlic is higher for those with allyl substituents and increased with increasing number of tethering sulfur atoms (103). Another study found that DATS treatment can significantly increase the H_2_S level, reduce the infarct size, and preserve cardiac function in mice after myocardial ischemia-reperfusion. This study substantiated the notion that DATS may be cardioprotective via H_2_S-related pathway (104).

Besides these polysulfide H_2_S donors, another group of organosulfur compounds that exert cardioprotective effects through H_2_S mediated pathways are cysteine derivatives, including SAC and its synthetic analog *S*-propargyl cysteine (R)-2-amino-3-(2-propynylthio) propanoic acid, SPRC. Instead of releasing H_2_S by themselves or by reacting with other compounds, they are believed to function by mediating endogenous H_2_S production. Increased CSE gene expression, elevated plasma H_2_S level and decreased mortality, infarct size, and ventricular hypertrophy were observed in acute myocardial infarction mice treated with SAC (105) or SPPC (106). The abolishment of these beneficial effects by a CSE inhibitor propargylglycine substantiated that their cardioprotective functions are H_2_S dependent.

Although many organosulfur compounds have been found from dietary source, only a few of them have been studied for H_2_S releasing activity. From a chemical point of view, polysulfides with more than two tethering sulfur atoms should be able to release H_2_S through thiol-disulfide exchange with GSH. Research on the H_2_S releasing activity by these compounds might provide new explanations for their purported cardiovascular benefits, and, therefore, warrants in-depth investigation.

## Organopolysulfides as Reactive Oxygen Species Scavengers

The implication of reactive oxygen species (ROS) in the development of CVDs has been shown in numerous studies in the past decades. The most important ROS in cardiovascular system are superoxide anion radical ($O_{2}^{•-}$) and hydrogen peroxide (H_2_O_2_). $O_{2}^{•-}$ is produced in vascular cells by a wide variety of oxidases, including the predominant NADPH oxidases, as well as lipoxygenases, xanthine oxidase, cytochrome P450, uncoupled mitochondrial electron transfer chain, and uncoupled endothelial nitric oxide synthase (eNOS) (107). $O_{2}^{•-}$ undergoes dismutation by superoxide dismutase (SOD) to generate H_2_O_2_. Superoxide anion rapidly would react with NO to generate peroxynitrite (ONOO^−^). This reaction consumes NO and reduces it activity in maintaining healthy cardiovascular functions. On the contrary, the resulting product, ONOO^−^, is an important lipid oxidation mediator that will lead to the oxidation of low-density lipoprotein (LDL), forming strong proatherogenic oxidized LDL (ox-LDL) (108). Besides, ROS contributes to cardiovascular injury through the modulation of multiple cellular responses, including monocyte adhesion, platelet aggregation, vascular smooth muscle cell apoptosis, migration and proliferation, inflammatory gene expression, and dysfunction of endothelium dependent relaxation (109).

Sulfur atoms in dietary organosulfides are electron rich and ready to donate electrons in a redox reaction; therefore, they are supposed to be good oxidant scavengers. AGE and garlic oil containing high amounts of organosulfurs have been shown to scavenge ROS and prevent damage caused by oxidative stress (110–113). However, very limited reports studied the scavenging activities of individual organosulfur compounds to certain types of free radicals. Those reported works are inconsistent and incomparable likely because different evaluating methods and sample concentrations were employed. In addition, nearly all of the studies were *in vitro* tests carried out in simple chemical systems because of the lack of analytical methods that can selectively target a specific free radical *in vivo*. But the general trend is, SAC, the water soluble, major organosulfur compound in AGE, possess strong scavenging activities against peroxynitrite (114, 115) and hydroxyl radical (114–116), but has negligible effects toward $O_{2}^{•-}$ or H_2_O_2_ (114, 116). However, the major lipophilic polysulfides in garlic oil DADS and DATS can inhibit $O_{2}^{•-}$ as much as SOD/ascorbic acid can (114) and their activity increases with the number of sulfur atoms; besides, they are good ONOO^−^ scavengers but have very limited effects on hydroxyl radical^.^or H_2_O_2_ (114). The potent hypochlorite acid scavenging effect of SAC, DADS, and DATS as well as some other thioallyl compounds such as allyl mercaptan, allyl methyl sulfide, dipropyl sulfide, and allicin has been reported in by several studies (115, 117, 118). Allicin is a good scavenger against $O_{2}^{•-}$, OH^⋅^, and ONOO^−^ (116, 117), but their activity seems to be attributed to the sulfenic acid formed from the copper elimination of allicin (119).

The cardioprotective effects of organosulfur compounds against oxidative damage in cell lines or in animals have been shown in several studies. SAC, which was able to inhibit LDL oxidation, was also reported to dose-dependently inhibit the H_2_O_2_ formation in ox-LDL challenged human umbilical vein endothelial cells (HUVEC) (120). Similar protective effect was also found in bovine pulmonary artery endothelial cells (121). DATS and DADS are reported to decrease the cellular peroxide level in ox-LDL-treated HUVEC cells by as much as 50 and 43%, respectively (122). DATS was shown to decrease the ROS and $O_{2}^{•-}$ levels in H9c2 cells induced by high-glucose treatment, and protect cardiac myocytes from apoptotic cells death in culture medium as well as in diabetic rats (123) through ROS related pathways. Similar effects were also found in rats treated with DADS and garlic oil (124). However, it needs to be pointed out that these protective effects against ROS damage observed in cell lines or in experimental animals might come from the combination of a wide range of physiological pathways such as enhancement of antioxidant enzyme expression or inhibition of peroxidation enzymes production activity, instead of direct free radical scavenging.

## Enzyme Activation/Inactivation and Gene Regulation

**Figure 7:**
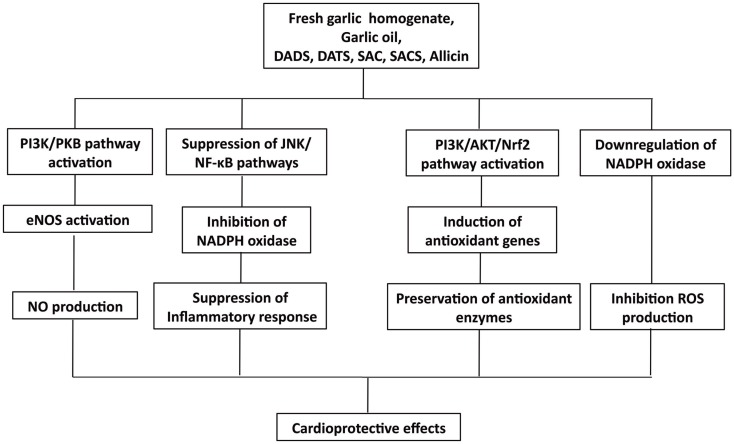
**Overview of cardioprotective effect of organosulfides via enzyme and gene regulations**.

Recently, a number of studies have suggested that the beneficial effects of organosulfides in cardiovascular health are associated with their ability to modulate antioxidant genes and enzyme expression (Table 3). In this section, we discuss these mechanisms and present an overview of the important biochemical pathways associated in the cardioprotective effects of organosulfides (Figure 7).

**Table 3:** ***In vivo* and *in vitro* studies showing the effect of organosulfides on enzyme and gene regulation and the underlying mechanisms**.

Organosulfide	Study	Enzyme/Gene	Experimental object	Dose	Organosulfide preparation	Mechanism	Reference
DATS	*In vitro*	Antioxidant enzymes (HO-1, SOD-1, SOD-2)	H9c2 high-glucose cardiomyoblast cells	10 μM	Garlic oil (40% DATS)	Upregulate the P13K/Ak/Nrf2 pathway; activate antioxidant enzyme system	(125 )
	*In vivo*	Genes (*HO-1*, *SOD-2*, *yGCS*)	Streptozotocin-induced diabetic male Wistar rats	
Allicin, alliin, *S*-allyl-l-cysteine, deoxyalliin, vinyldithiin	*In vivo*	Myocardial catalase, myocardial SOD, glutathione peroxidase (GPx) *MnSOD* gene	Sprague-Dawley rats	250 mg^−1^kg^−1^day^−1^	Raw garlic homogenate	Activation of endogenous antioxidant defenses and reduction of oxidative stress and cardiac hypertrophy as a result of increased myocardial Nrf2 expression; Increased Mn-SOD expression, myocardial SOD, catalase, and GPx	(126 )
DADS, DATS	*In vivo*	*SOD-1* gene	Weanling male Wistar rats	40 mg^−1^kg^−1^ BW DADS and DATS; 100 mg^−1^kg^−1^ BW GO	Garlic oil and pure compounds	Increased expression of *SOD-1* preserved antioxidant action against oxidative stress during diabetic cardiomyopathy	(124 )
DATS	*In vivo*	Superoxide dismutase (SOD), glutathione peroxidase (GPx)	Obese diabetic rat; high-glucose-induced endothelial cell	Animal: 5.0 mg^−1^kg^−1^day^−1^ Cell: 25-100 μmol/L	Pure compound	Elevated the activities of SOD and GSH-Px in mitochondrium	(127 )
DATS	*In vitro*	c-Jun N-terminal kinases (JNKs)	H9c2 cardiomyoblast cells; neonatal cardiomyocytes	1–10 μM	Garlic oil (40% DATS)	Suppression of ROS-stimulated downstream JNK/NF-κB signaling	(123)
DATS, DADS	*In vitro*	Endothelial NOS (eNOS)	LDL-treated HUVEC	200 μM DADS 50 μM DATS	Pure compounds	Preserved the interaction of eNOS with calveolin-1; suppressed the reduction of the cellular eNOS protein by ox-LDL	(128 )
Not specified	*In vivo*	Myocardial catalase, GPx, mitovchondrial enzymes i.e., citrate cynthase and B hydroxyacyl CoA dehydrogenase	Male Swiss albino mice	250–500 mg^−1^ kg^−1^day^−1^	Saline and aqueous garlic homogenate	Garlic homogenate preserved expression of antioxidant enzymes and attenuated isoproterenol-induced cardiac changes. GO preserves activity of antioxidant defense enzymes and their interaction with NO pathway	(129 )
DATS	*In vivo*	eNOS	MI/R mice model	200 μ/g	Pure compound	Activates eNOS and improved NO bioavailability	(104 )
Allicin	*In vivo*	SOD	Wistar rats	6–10 mg^−1^ kg^−1^day^−1^	Pure compound	Increase in SOD helps inhibit lipid peroxidation by hyperhomocysteinemia and regulates the excretion and balance of plasma endothelin and NO	(130 )
SACS	*In vivo*	SOD, catalase	Female Wistar albino rats	0.111–0.222 mg^−1^ kg^−1^ SACS; 125–250 mg^−1^ kg^−1^ garlic homogenate	Fresh garlic homogenate and pure compound	Restoring SOD and catalase to normal levels	(131 )

### Nrf2 activation and antioxidant gene modulation

Oxidative stress, viewed at the molecular level, is linked to the activities of nuclear factor-E-2-related factor (Nrf2) in the nucleus, which upregulates genes that encode for the expression of antioxidant enzymes (132). It has been demonstrated that Nrf2 coordinates the expression and upregulation of various antioxidant enzymes, including heme oxygenase-1 (HO-1), glutathione *S*-transferase (GST), and SOD (133, 134). These enzymes collectively protect cardiomyocytes from oxidative stress (135, 136). For example, SOD-1, one of the three SOD, works together with catalase to detoxify superoxides and hydrogen peroxide (H_2_O_2_) (137). Induced upregulation or downregulation of *SOD-1* genes and expression of the antioxidant enzyme SOD-1 are often evaluated as an indicator of ROS stress.

The induced Nrf2-mediated antioxidant gene activation by DATS is linked to several pathways, including MAPK, PKC, and PI3K/AKT (138). Tsai et al. showed that the cytoprotective effect of DATS against oxidative stress in high-glucose exposed neonatal cardiomyocytes and streptozotocin-induced diabetic rats is by activation of the P13K/Akt/Nrf2 pathway (125). Expression of Nrf2 proteins was upregulated by DATS in a time-dependent manner resulting in a dose-dependent improvement of the expression of antioxidant genes, including *HO-1* and *SOD2*. Moreover, PI3K-specific SiRNA and Nrf2-specific SiRNA transfected cells showed normal levels of superoxide than those administered with DATS alone, indicating that the antioxidative effect of DATS is suppressed by Nrf2 and PI3K SiRNA, further suggesting that the cytoprotective effect of DATS against high-glucose-induced oxidative stress is by the activation of P13K/Akt/Nrf2 pathway (125). The ability of DATS to induce Nrf2 activation was further demonstrated in a study that specifically looked at Keap1 cysteine 288 residue, one of the cysteine residues that is essential in regulating Nrf2 activation (139). It was found that mono-allyl mono-sulfide may attach to the Keap1 peptide fragment containing Cys288, indicating that DATS specifically interacts with this cysteine residue accounting for its ability to induce Nrf2 activation (139). Recently, raw garlic homogenate administration to fructose fed-diabetic rats showed increased myocardial Nrf2 expression, along with increased Mn-superoxide dismutase (*Mn-SOD*) gene expression and, elevated myocardial SOD, catalase, and glutathione peroxidase (GPx) activities (126). In addition to enhanced antioxidant gene and enzyme expressions, this study also showed that garlic homogenate can increase the levels of phospho-PI3K and phospho-AKT, indicating that PI3K/AKT pathway plays a major role in cardiac hypertrophy and oxidative stress by activating Nrf2, which, in turn regulates the activation of antioxidant defense enzymes during cardiovascular complications (126). In another study, administration of garlic oil and pure compounds of DADS and DATS increased level of SOD-1 expression and enhanced the PI3K/AKT signaling in diabetic rats (124).

### Modulation of endothelial nitric oxide synthase

Oxidized LDL is an important factor in the pathogenesis of atherosclerosis (108). Ox-LDL promotes vascular dysfunction in various mechanisms, one of which is its inhibition of eNOS activity resulting in the alteration of the NO-regulated responses in the endothelial cells (140, 141). Organosulfur compounds from garlic are involved in the modulation of eNOS activity and are responsible for garlic’s anti-atherogenic effect. Lei et al. studied garlic’s role against ox-LDL in HUVEC and found that the protective role of DADS and DATS in eNOS activation and NO production is associated with the PI3K/PKB pathway (128). Ox-LDL was found to decrease PKB and eNOS phosphorylation but this effect was abolished by DADS and DATS pretreatment along with restored production of NO. Moreover, treatment with PI3K inhibitor wortmanin attenuated the protective effect of DADS and DATS in eNOS activation and recovery of NO production (128). Serine phosphorylation and blocking of eNOS activation may result from the inhibition of PI3K/PKB pathway or from PKB site mutation on the eNOS protein (at serine 1177) signifying the importance of PI3K/PKB pathway in regulating eNOS activity (142). In another study, garlic homogenate (250 mg kg^−1^ day^−1^ for 30 days) treatment on isoproterenol-induced myocardial infarction mice model relieved oxidative stress by significantly increasing release of GPx and catalase activities (129). This study also demonstrated the positive association of NOS activation, NO production, and antioxidant enzyme activity protection with garlic treatment suggesting that the maintenance of redox balance through protecting the activation of NOS and, hence, production of NO, explain garlic’s protective role against myocardial damage. Moreover, eNOS activation was demonstrated by Predmore et al., showing that DATS treated rat myocardial tissue had an increased eNOS phosphorylation at Ser^1177^ and elevated levels of NO metabolites, including nitrite and nitrate (104).

### Modulation of antioxidant enzymes and inhibition of NADPH oxidase activity

The protective effect of DATS against hyperglycemia-induced oxidative stress was demonstrated in an *in vivo* model of obese diabetic rats and in high-glucose-treated endothelial cells (127). The reduction of mitochondrial oxidative stress was suggested to be the action mechanism because attenuation of endothelial cell impairment was observed along with enhanced activities of SOD and GPx in the mitochondria upon administration of DATS. SOD and GPx are antioxidative enzymes with detoxifying actions against mitochondrial ROS (143). DATS attenuated the hyperglycemia-induced NADPH oxidase and its related ROS production in the mitochondria mainly by preserving but not upregulating the activities of SOD and GPx (127). In another study, the effect of low (6 mg kg^−1^ day^−1^) and high (10 mg kg^−1^ day^−1^) allicin doses was demonstrated in hyperhomocysteinemia-induced vascular endothelial dysfunction animal model (130). Hyperhomocysteinemia is another cause of oxidative stress that may lead to vascular endothelial dysfunction and injury (144, 145). Formation of H_2_O_2_ may result from the self-oxidation of homocysteine at the active free sulfhydryl group. Homocysteine level in rats with hyperhomocysteinemia was reduced by treatment with allicin along with an increase in SOD activity (130). Similar results were demonstrated in another study employing fructose-induced hypertensive rat models (131). SOD and catalase were restored to normal levels after treatment with *S*-allyl cysteine sulfoxide (SACS) isolated from fresh garlic homogenate.

In a recent paper, expression of p22phox and gp91phox, the subunits comprising the membrane-bound component cytochrome b_558_, and the subunits responsible for the activity of NADPH oxidase increased along with the production of superoxide free radicals ($O_{2}^{•-}$) in high-glucose-treated H9c2 cells but was attenuated by treatment with DATS (1–10 μM). Production of ROS by NADPH oxidase is related to the activation of c-Jun N-terminal kinases (JNK) signaling and the transcriptional factor NF-κB (146). Activation of JNK leads to their translocation into the nucleus where they phosphorylate transcription factors including c-Jun and p53 that are involved in the regulation of apoptosis (147, 148). Treatment with DATS (1–10 μM) dose-dependently inhibited the high-glucose activation of JNK, which was suggested to be associated with the inhibition of NADPH oxidase-regulated ROS generation in H9c2 cells and neonatal primary cardiomyocyte (123). Treatment with DATS (1–10 μM) inhibited the nuclear translocation of NF-κB in H9c2 cells and reduced the protein levels of NF-κB in streptozotocin-induced diabetic rats (40 mg/kg BW DATS) (123). Hyperglycemia is not only known to induce ROS generation but also inflammation, which could activate transcription regulators, including NF-κB, which, in turn, regulates intracellular apoptosis (149, 150). Treatment with DATS protects high-glucose-treated neonatal cardiomyocytes and H9c2 cells from ROS damage by inhibiting the activation of JNK/NF-κB pathways (123).

## Conclusion

**Figure 8:**
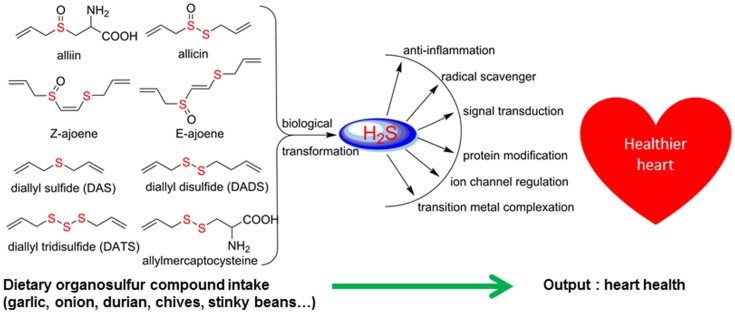
**Hydrogen sulfide as the common denominator for bioactivity of dietary organosulfur compounds**.

There are significant scientific research papers suggesting that dietary organosulfurs have broad range of bioactivity including cancer chemoprevention and promotion of cardiovascular health. While there is strong evidence to suggest that isothiocyanates may be the active form in cruciferous vegetables for their cancer chemopreventive property, the daily intake of such compounds could be very low because cooking of these vegetables will prevent their formation. For other organosulfides, it remains a challenge to pinpoint the compounds responsible for their purported health promoting effects. Allicin was considered as the active components of garlic and has been used as a marker compound in garlic supplement standardization. The null results from clinical trials make us reconsider other potential compounds. The discovery of DATS and DADS as donors of hydrogen sulfide opens up a new avenue for establishing evidence of organosulfide action mechanisms on promoting cardiovascular health. It remains to be seen whether H_2_S donating activity can be the unifying mechanism for dietary organopolysulfides to exert their health benefits. H_2_S is a “double-edged sword” as it is toxic at high concentrations. Rapid burst of H_2_S from organosulfides may lead toxic effects *in vivo* but it may be needed for their anti-microbial activity. For cardiovascular health, slow (or controlled) release of H_2_S from dietary polysulfides would be desired. There are many other organosulfides in our diet that have not been investigated yet. The outstanding ones include cyclic polysulfides founds in stinky beans and mushrooms. While these polysulfides are important flavoring molecules (sometime smelly!), little is known on their health promoting activity. The high sulfur loading of them (i.e., compounds shown in Figure 3) would make them ideal reservoirs of H_2_S, if they can be biotransformed to release H_2_S in human body. Research work in the future shall be focused on establishing the structure and H_2_S releasing potentials and rates of individual dietary polysulfides in cell line and animal model systems. Since processing conditions can greatly alter the polysulfide profiles in foods, an H_2_S releasing activity guided optimization of processing conditions would lead to optimal effectiveness of supplements or functional foods based on *Alliums* and stinky beans for their cardioprotective effects (Figure 8). The research is just at the beginning on taming the pesky dietary organosulfides for human health promoting and disease prevention.

## Conflict of Interest Statement

The authors declare that the research was conducted in the absence of any commercial or financial relationships that could be construed as a potential conflict of interest.
